# Patient needs four years after first psychiatric hospitalization in a Brazilian cohort

**DOI:** 10.1590/1414-431X2021e11447

**Published:** 2021-07-23

**Authors:** M.E.S.B. Santos, D.L. Roza, R.E.M. Barros, J.L.F. Santos, D. Razzouk, J.M. Azevedo-Marques, P.R. Menezes, C.M. Del-Ben

**Affiliations:** 1 Departamento de Neurociências e Ciências do Comportamento, Faculdade de Medicina de Ribeirão Preto, Universidade de São Paulo, Ribeirão Preto, SP, Brasil; 2 Departamento de Medicina Social, Faculdade de Medicina de Ribeirão Preto, Universidade de São Paulo, Ribeirão Preto, SP, Brasil; 3 Centro de Economia da Saúde Mental, Departamento de Psiquiatria, Universidade Federal de São Paulo, São Paulo, SP, Brasil; 4 Departamento de Medicina Preventiva, Faculdade de Medicina, Universidade de São Paulo, São Paulo, SP, Brasil; 5 Núcleo de Pesquisa em Saúde Mental Populacional, Universidade de São Paulo, São Paulo, SP, Brasil

**Keywords:** Needs assessment, Mental disorders, Cohort, Low- and middle-income countries, Brazil

## Abstract

Knowledge about the needs of psychiatric patients is essential for mental health care planning. However, research on met and unmet needs is still scarce, particularly in low- and middle-income countries. This study aimed to describe the patients' needs (met and unmet) at least four years after their first psychiatric hospitalization and to verify the role of demographic and clinical features as possible predictors of these needs. Patients who had their first psychiatric admission between January 1, 2006 and December 31, 2007 at an inpatient unit in the city of Ribeirão Preto, Brazil, were eligible to participate in the study. Patients were contacted and face-to-face interviews were conducted by psychologists using the Camberwell Assessment of Need. Data were analyzed using zero-inflated negative binomial regression model. Of 933 eligible patients, 333 were interviewed. The highest level of needs was related to welfare benefits (32.4%, unmet=25.5%), followed by household skills (30.3%, unmet=3.0%), psychotic symptoms (29.4%, unmet=9.0%), psychological distress (27.6%, unmet=8.4%), physical health (24.3%, unmet=5.4%), daytime activities (19.5%, unmet=16.5%), and money (16.8%, unmet=9.0%). Fewer years of schooling, living with relatives, and unemployment at the moment of the first admission were significantly associated with a higher number of both met and unmet needs in the follow-up. Unmet needs were also more often reported by patients living alone. In conclusion, socioeconomic indicators were the best predictors of needs. The unmet needs related to welfare benefits point to the need for specific social and health policies.

## Introduction

In Brazil, the high prevalence of mental disorders (1-3) contrasts with the mental health expenditure, which is less than 1% of the national health budget (4), with the implementation of effective community-oriented mental health care still an ongoing process (5). Thus, identifying of possible barriers to appropriate treatment for each person and specific population groups is an important step to guide the application of scarce resources (6).

A way of evaluating if people are receiving proper care for their problems is assessing their needs, which can be defined as “the requirements of the individual to enable him/her to achieve, maintain, or restore an acceptable level of social independence or quality of life” (7). A comprehensive assessment of needs provides relevant information that allows to develop personalized care plans and evaluate the ability of health services in providing effective care (8-10), which are particularly important for people with mental disorders, that frequently also present non-psychiatric diseases and social barriers for adequate care (11,12).

Among people with mental disorders, higher rates of needs seem to be related to the male sex, unemployment, lower economic level (13,14), diagnosis of severe mental disorders (affective disorders, schizophrenia, and substance abuse-related disorders) (15,16), psychotic symptoms severity (17), and more frequent contact with community-based mental health services (18). There is also an association between unmet needs and worse quality of life for these people (19), with the reduction of unmet needs predicting an improvement of the quality of life (18). Moreover, mental health services' effectiveness is directly related to the satisfaction of the patient's needs (19).

The premise that mental health care should be offered based on the patients' needs has been adopted by different countries (20,21), but few studies have been carried out in low- and middle-income countries (LMICs), where the needs can have a different profile compared to those reported in wealthy countries. A study compared the needs of patients in five European countries (20) and found that the mean number of needs in each country ranged from 6.3 to 4.8, and the most frequently reported unmet needs were related to company, intimate relationships, and psychological distress. In LMICs, countries such as Kuwait, India, and Brazil, the most frequently reported unmet needs were related to money, education, accommodation, and information (2,22-24), which seem to be more associated with possible socioeconomic disadvantages than with the severity and prognosis of the mental disorder itself.

To improve the quality of the information about patients' needs that can be used for the mental health care policies in Brazil and other LMICs, we assessed the needs of a cohort of Brazilian patients who had a mental disorder severe enough to justify a first psychiatric admission. As far as we know, this is the first Brazilian study assessing the needs of a representative sample of adult people hospitalized for the first time in psychiatric wards, and not just in a single service or with a specific diagnostic category. This study aimed to describe the patterns of patient's met and unmet needs after at least four years from their first psychiatric admission and to verify the role of demographic and clinical features and use of mental health services as possible predictors of those needs. We hypothesized that Brazilian patients have a higher number of needs – particularly unmet needs – than patients from wealthier countries.

## Material and Methods

### Context of the study

This study was conducted in Ribeirão Preto, state of São Paulo, Brazil. In the last demographic census (2010), Ribeirão Preto's population was estimated at 604,682 inhabitants, with a Human Development Index (IDH) score of 0.800, a gross internal product equivalent to US$7,827,227, and a per capita monthly income of US$730. Ninety-nine percent of the population lived in the urban area (http://www.cidades.ibge.gov.br/).

The public mental health services network in the Ribeirão Preto catchment area has been described previously (25). Briefly, it is a regionalized system, with each community-based service being responsible for a specific catchment area, regardless of the patient's diagnosis or severity of the mental disorder. The inpatient units are closely connected with each other and with community-based services. When this study was conducted, Ribeirão Preto shared with twenty-five cities in the region (estimated population of 1,327,989 inhabitants) 108 psychiatric beds: six in an emergency hospital, 22 beds in psychiatric wards in a general hospital, and 80 beds in a psychiatric hospital.

### Study design and protocol

This was a cohort study based on a database of all psychiatric admissions (24 h or more in a hospital's bed) that occurred in the Ribeirão Preto catchment area over 10 years (1998-2007) (25,26). For this study, we included the patients who lived in the city of Ribeirão Preto and had their first psychiatric admission between January 1, 2006 and December 31, 2007, in one of the inpatient psychiatric units described above.

At least four years after the discharge from the first psychiatric admission, attempts were made to locate and contact all patients based on addresses and telephone numbers available in the original database and the public health system. The attempts to contact patients were made in chronological order, according to the date of the first psychiatric admission. Data collection took place between December 1, 2010 and December 31, 2011.

Numerous methods were used to contact the patients: telephone calls, invitation letters sent by mail, and home visits. All available sources of information were used, including relatives and neighbors. For each possible participant, at least three contact attempts were made before the participant was considered as missing. Home visiting proved to be the most fruitful method for reaching patients and collecting data.

### Data collection

Data regarding the first admission were extracted from the original database. In the follow-up assessment, participants were assessed by the Camberwell Assessment of Need – Research version (CAN-R) scale (8), translated into Portuguese with very good reliability and validity indexes (27). The CAN-R evaluates 22 domains of needs of formal and informal care: accommodation, food, household skills, self-care, daytime activities, physical health, psychotic symptoms, information, psychological distress, safety to self, safety to others, alcohol, drugs, company, intimate relationships, sexual expression, childcare, basic education, telephone, transport, money, and welfare benefits. For each domain, needs were categorized in: a) “Absent” (no serious problem); b) “Met need” (no serious problem or moderate problem because of continuing intervention/care); and c) “Unmet need” (current serious problem). Additional information regarding the use of mental health services (current treatment and occurrence of readmission) was also collected using a semi-structured questionnaire designed specifically for this study.

Two psychologists trained to conduct interviews with psychiatric patients and not involved in the mental health care of the respondents carried out the interviews. The interviewers' training consisted of: a) joint reading of the instruments, followed by discussions about the features and rules of application of the instruments and procedures for collecting data; b) joint interviews conducted by the first author followed by discussion until consensus was reached for at least 70% of the responses; and c) monthly meetings aimed at clarifying and resolving questions regarding the application of the tools.

### Definition of variables

The main outcome of this study was patient needs after a follow-up period of at least four years after their first psychiatric admission, measured by: a) the total number of needs (met + unmet); and b) the frequencies of met and unmet needs of each domain of the CAN-R.

The sociodemographic variables included were those recorded at the first admission, and categorized as follows: a) sex: women, men; b) age: less than 29 years old, 30 to 49 years, 50 years or more; c) marital status: with (married or cohabiting), without a partner (single, separated, divorced, or widowed); d) self-reported skin color: white, non-white (brown, mixed, black); e) years of education: up to 8 years, 9 years or more; f) household: relatives, partner, alone; and g) employment: yes, no.

Considering the variety of diagnoses, the main psychiatric diagnoses recorded at the discharge of the first admission were distributed in five groups, as follows: a) psychoactive substance-related disorders (ICD-10=F10-F19); b) psychotic disorders (F20-F29); c) mood disorders (F30-F39); d) non-psychotic disorders (F40-F69); and e) miscellaneous/others: (F00-F09 and F70-F99).

For the characterization of the services’ use we considered: a) the unit where the first psychiatric admission occurred: emergency unit, general hospital, psychiatric hospital; b) the length of stay during the first admission: 1 to 3 days, 4 to 30 days, or 31 days or longer; c) current follow-up in a community-based service at the moment of the follow-up interview: yes, no; and d) occurrence of inpatient readmission during the follow-up period: yes, no.

### Statistical analysis

We built frequency tables for the analysis of demographic and clinical features, and of met and unmet needs. To verify the association between variables, the Pearson's chi-squared test was used. For the quantitative variables, the descriptive measures are reported as means and standard deviation (SD). To explore the possible predictors of needs (met and unmet), a zero-inflated negative binomial (ZINB) regression model was used. A particular challenge was that a large number of respondents reported having no need. A closer inspection of the data indicated that the dependent variable, number of needs, contained an excessive number of zeros and an overdispersion. Counting data methods such as Poisson or negative binomial distributions are not directly suitable for analysis in the case of excess of zeros. Our approach was to try regression models, zero-inflated Poisson (ZIP), and ZINB. The Akaike information criterion (AIC), likelihood ratio test, and Vuong test were used to select the best model. ZINB regression model with log link function was estimated using maximum likelihood estimate for the parameters. The residual of the models was verified by the Pearson and deviance residuals. Two models were built. In model 1, we performed a simple analysis, the number of needs as the dependent variable and each of the demographic and clinical variables as independent variables. In model 2, we performed a multiple analysis, including the variables with a P-value lower than 0.05 in model 1 as the independent variables and the number of needs as the dependent variable. The collinearity diagnostics in the models was verified with the variance inflation factor (VIF). To aid in the interpretation of the results, the regression coefficients were exponentiated and presented as rate ratios (RRs). The RR based on the regression coefficients is reported with 95% confidence intervals (CIs). The significance level was set at 0.05. Statistical analyses were performed using R software version 3.6.3 (R Core Team; https://www.R-project.org/) and SAS software version 9.4 for Windows (SAS Institute Inc., USA).

### Ethical approval

The local ethics committee approved the study (research protocol number 10184/2009), and all participants and their relatives, in the case of psychotic patients, signed an informed consent form.

## Results

### Features of patients at first admission

Nine hundred and thirty-three patients from Ribeirão Preto were registered in the database (25) with the first psychiatric admission during the 2006-2007 period and considered eligible for this study. Of all, 523 (56.1%) were male, with a mean age of 36 years (SD=14.2), ranging from 11 to 88 years. More than half of the patients were admitted to the emergency unit (n=482, 51.7%), followed by the psychiatric hospital (n=328, 35.1%) and the general hospital (n=123, 13.2%). By the time of their first admission, 74.8% of patients were unemployed, and 69.2% were single. The most frequent diagnosis was substance use disorders (F10-F19; 31.4%) (Table 1).

**Table 1:** Associations between the sociodemographic characteristics of the interviewed and non-interviewed patients.

	Non-interviewed N=600 (64.3%)	Interviewed N=333 (35.7%)	All N=933	P-value (chi-squared test)
Sex				
Men	243 (40.5)	167 (50.1)	410 (43.9)	<0.01
Women	357 (59.5)	166 (49.9)	523 (56.1)	
Age (years)				
≤29	245 (41.1)	81 (24.3)	326 (35.1)	<0.01
30-49	268 (45.0)	165 (49.6)	433 (46.6)	
≥50	83 (13.9)	87 (26.1)	170 (18.3)	
Marital status				
Without a partner	434 (72.5)	211 (63.4)	645 (69.2)	<0.01
With a partner	165 (27.5)	122 (36.6)	287 (30.8)	
Employment				
No	477 (79.6)	207 (65.7)	684 (74.8)	<0.01
Yes	122 (20.4)	108 (34.3)	230 (25.2)	
Diagnosis (ICD-10)				
Miscellaneous/Others (F00-F09; F70-F99)	34 (5.7)	18 (5.4)	52 (5.6)	<0.01
Substance-related disorders (F10-F19)	215 (35.8)	78 (23.4)	293 (31.4)	
Psychotic disorders (F20-F29)	108 (18.0)	63 (18.9)	171 (18.3)	
Mood disorders (F30-F39)	170 (28.3)	114 (34.2)	284 (30.4)	
Non-psychotic disorders (F40-F69)	73 (12.2)	60 (18.1)	133 (14.3)	
Inpatient unit				
Psychiatric hospital	211 (35.2)	117 (35.1)	328 (35.1)	0.23
General hospital	71 (11.8)	52 (15.6)	123 (13.2)	
Emergency unit	318 (53.0)	164 (49.3)	482 (51.7)	
Length of stay (days)				
1-3	321 (53.5)	176 (52.9)	497 (53.3)	0.98
4-30	233 (38.8)	131 (39.3)	364 (39.0)	
≥31	46 (7.7)	26 (7.8)	72 (7.7)	

### Follow-up

**Figure 1:**
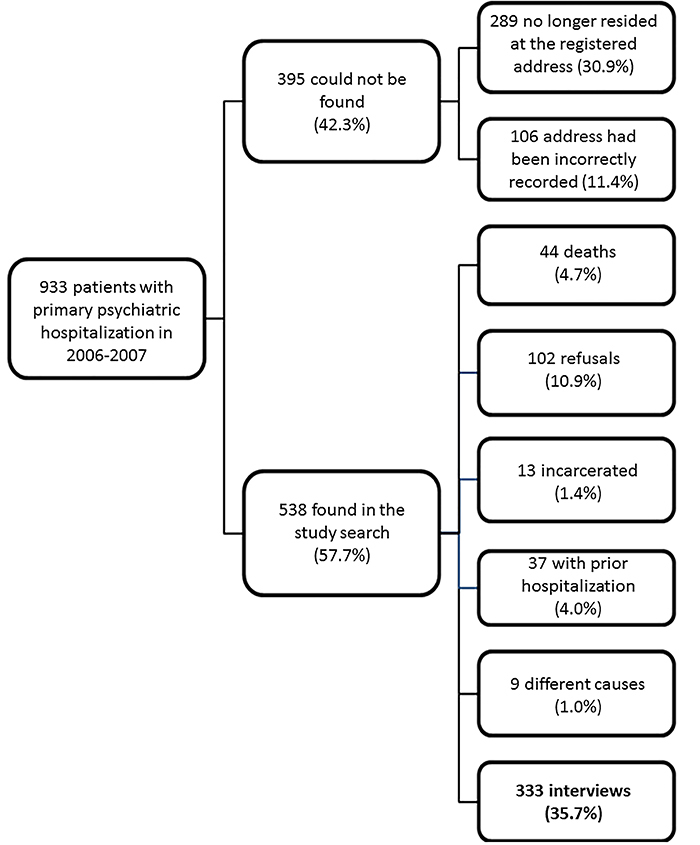
Flowchart of the patients enrolled in the study.

After a mean period of 53.5 months (SD=3.59, ranging from 48 to 59 months), from a total of 933 patients, 395 (42.3%) could not be found: 289 (30.9%) because they no longer resided at the registered address and 106 (11.4%) because the addresses had been incorrectly recorded in the database. As Figure 1 shows, communication was made with 538 (57.7%) patients or their relatives, but face-to-face interviews for data collection were not held for another 205 (22.0%) participants: 102 (10.9%) refused to participate in the study; 13 (1.4%) were incarcerated at the time of the interview; 44 (4.7%) had died according to information provided by relatives; 37 (4.0%) had already been admitted to a psychiatric hospital before the hospitalization reported in the database; and 9 (1.0%) were excluded for other reasons, such as having taken an extended trip, disappeared, or had been admitted for treatment (e.g., residential treatment for drug addiction) outside of Ribeirão Preto.

Three hundred thirty-three individuals (35.7% of the initial sample of first-admission patients) were interviewed. Compared with patients assessed by face-to-face interviews, the missing patients were younger, predominantly female, single, unemployed, and mainly with substance-related mental disorders. There were no significant differences in terms of length of stay and inpatient unit (Table 1).

### Demographic and clinical features of the patients assessed in the follow-up

The distribution was homogeneous between sexes, with 167 (50.1%) men and 166 (49.9%) women. Around half of the sample consisted of adults aged between 30 to 49 years (49.6%), with a few years of education (48.9%). The majority of the patients reported being white (55.9%), without a marital bond (63.4%), with some occupational activity (65.7%), and living with relatives (69.7%).

Patients were more often diagnosed with mood disorders (34.2%), followed by substance-related disorders (23.4%) and psychotic disorders (18.9%). The diagnoses of mood disorders were more common among women than men, whereas substance-related disorders were more often diagnosed among men than women.

More than half of the patients were being followed up in community-based mental health services at the moment of the assessment. Readmission during the follow-up period occurred in 22.2% of the patients.

### Needs

**Figure 2:**
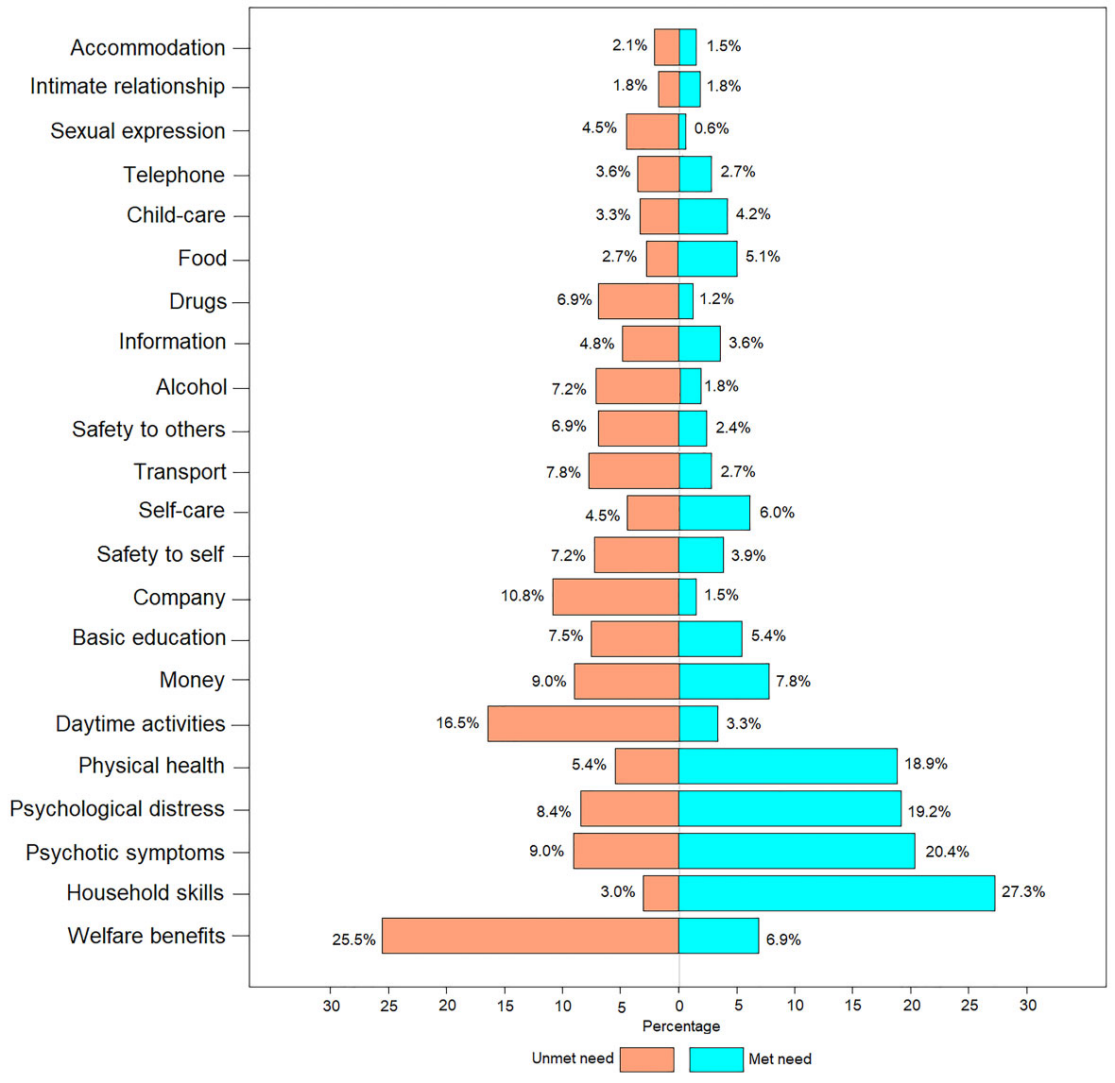
Bar plot of the distribution of needs (met and unmet) in each of the 22 domains of Camberwell Assessment of Need - Research version (CAN-R) for the 333 interviews (in percentage).

Considering the total sample, the mean number of needs was 3.07 (SD=3.10; unmet needs=1.59, SD=2.24; met needs=1.48, SD=1.72) and the number of needs per participant ranged from 0 to 15. Considering the distribution of needs of the 22 domains of the CAN-R (Figure 2), welfare benefits were the most critical unmet need reported. The subsequent unmet needs more often reported by the patients were related to daytime activities, company, drugs, alcohol, transport, safety to others, and sexual expression. The met needs more often reported by the patients were related to household skills, physical health, psychotic symptoms, and psychological distress.

### Zero-inflated negative binomial regression models

The ZINB regression model (Table 2) showed that five predictors were significantly associated with higher needs (met + unmet) at the follow-up (Model 1). There was a significant increase in the number of needs in patients with few years of schooling (RR=1.62; 95%CI: 1.29-2.04), indicating the expected number of needs for those with up to 8 years of study was 1.62 times higher the expected number of needs for those with 9 years of study or more. There was also a significant increase in the number of needs in patients who were, at the moment of the first admission, living with relatives (RR=1.41; 95%CI: 1.09-1.83), unemployed (RR=2.51; 95%CI: 1.98-3.17), admitted to a psychiatric hospital (RR=1.30; 95%CI: 1.01-1.68), and also psychiatric readmission during the follow-up (RR=1.38; 95%CI: 1.05-1.81). In model 2, two predictors related to health services’ use (admission to a psychiatric hospital and readmission in the follow-up period) were no longer significant; fewer years of education, living with relatives, and unemployment remained statistically significant.

**Table 2:** Predictors of the needs (met + unmet) of patients at least four years after their first psychiatric admission according to zero-inflated negative binomial (ZINB) regression model.

	N (%)	Model 1 [RR (95%CI)]	Model 2 [RR* (95%CI)]
Sex			
Men	167 (50.1)	1.13 (0.90; 1.43)	-
Women	166 (49.9)	1.00 (Reference)	
Age (years)			
≤29	81 (24.3)	0.94 (0.67; 1.30)	-
30-49	165 (49.6)	1.04 (0.78; 1.38)	-
≥50	87 (26.1)	1.00 (Reference)	
Marital status			
Without a partner	211 (63.4)	1.03 (0.81; 1.31)	-
With a partner	122 (36.6)	1.00 (Reference)	
Skin color			
White	186 (55.9)	1.15 (0.90; 1.46)	-
Non-white	131 (39.3)	1.00 (Reference)	
No information	16 (4.8)		
Education (years)			
≤8	163 (48.9)	**1.62 (1.29; 2.04)**	**1.48 (1.21; 1.81)**
≥9	170 (51.1)	1.00 (Reference)	1.00 (Reference)
Living arrangements			
Alone	29 (8.7)	1.44 (0.93; 2.24)	**1.56 (1.04; 2.32)**
With relatives	198 (59.5)	**1.41 (1.09; 1.83)**	**1.33 (1.05; 1.67)**
With partner	106 (31.8)	1.00 (Reference)	1.00 (Reference)
Employment			
No	207 (62.2)	**2.51 (1.98; 3.17)**	**2.34 (1.85; 2.96)**
Yes	108 (32.4)	1.00 (Reference)	1.00 (Reference)
No information	18 (5.4)		
Diagnosis (ICD-10)			
Miscellaneous (F00-F09 and F70-F79)	18 (5.4)	0.64 (0.37; 1.10)	-
Substance-related disorders (F10-F19)	78 (23.4)	1.02 (0.60; 1.74)	-
Psychotic disorders (F20-F29)	63 (18.9)	0.73 (0.42; 1.26)	-
Mood disorders (F30-F39)	114 (34.2)	0.69 (0.41; 1.15)	-
Non-psychotic disorders (F40-F69)	60 (18.1)	1.00 (Reference)	
Inpatient unit			
Psychiatric hospital	117 (35.1)	**1.30 (1.01; 1.68)**	0.96 (0.76; 1.21)
General hospital	52 (15.6)	1.29 (0.92; 1.80)	1.02 (0.76; 1.36)
Emergency unit	164 (49.3)	1.00 (Reference)	1.00 (Reference)
Length of stay (days)			
1-3	176 (52.9)	1.08 (0.69; 1.70)	-
4-30	131 (39.3)	1.12 (0.71; 1.78)	-
≥31	26 (7.8)	1.00 (Reference)	
Current follow-up			
No	139 (41.7)	0.93 (0.73; 1.18)	-
Yes	194 (58.3)	1.00 (Reference)	
Readmission			
Yes	74 (22.2)	**1.38 (1.05; 1.81)**	1.13 (0.89; 1.42)
No	259 (77.8)	1.00 (Reference)	1.00 (Reference)

Adjusted for education, living arrangements, employment, inpatient unit and readmission; RR = rate ratio; 95%CI: 95% confidence interval. Bold values indicate P<0.05.

The ZINB regression model separated by met needs (Supplementary Table S1) and unmet needs (Supplementary Table S2) were performed. Table 3 summarizes the significant results found in the multiple analyses for the two categories of needs. Two predictors were significantly associated with a greater number of met needs, fewer years of education (RR=1.36; 95%CI: 1.05-1.75), and unemployment (RR=1.90; 95%CI: 1.40-2.58); living alone (RR=0.50; 95%CI: 0.29-0.89) was associated with a lesser number of met needs (reduction of 50% compared to those living with a partner). Three predictors were associated with a greater number of unmet needs: fewer years of education (RR=1.61; 95%CI: 1.22-2.14), living alone (RR=2.74; 95%CI: 1.59-4.73) or with relatives (RR=1.64; 95%CI: 1.17-2.30), and unemployment (RR=2.45; 95%CI: 1.75-3.43).

**Table 3:** Predictors of the met and unmet needs of patients at least four years after their first psychiatric admission according to zero-inflated negative binomial (ZINB) regression model.

Predictors	Met need [RR (95%CI)]	Unmet need [RR (95%CI)]
Education (years)		
≤8	**1.36 (1.05; 1.75)**	**1.61 (1.22; 2.14)**
≥9	1.00 (Reference)	1.00 (Reference)
Living arrangements		
Alone	**0.50 (0.29; 0.89)**	**2.74 (1.59; 4.73)**
With relatives	1.21 (0.92; 1.59)	**1.64 (1.17; 2.30)**
With partner	1.00 (Reference)	1.00 (Reference)
Employment		
No	**1.90 (1.40; 2.58)**	**2.45 (1.75; 3.43)**
Yes	1.00 (Reference)	1.00 (Reference)

Only the significant predictors (P<0.05) are presented, according to model 2. The complete results of the met needs are available in Supplementary Table S1 and the unmet needs are available in Supplementary Table S2. Met need: adjusted for education, living arrangements, employment, inpatient unit, current follow-up and readmission; Unmet need: adjusted for education, living arrangements, employment, inpatient unit and readmission; RR: rate ratio; 95%CI: 95% confidence interval. Bold values indicate P<0.05.

Collinearity diagnostics in models did not indicate significant multicollinearity between the variables with VIF ranging from 1.02 to 1.28. The likelihood ratio test was significant (P<0.01), indicating the ZINB regression model had a better fit to the data over the ZIP regression model. Vuong test was significant (P=0.01), suggesting the ZINB regression model had a better fit to the data over a standard negative binomial regression model.

## Discussion

In this follow-up study, we verified the needs of individuals with mental disorders four years after their first psychiatric admission. Although variables related to the use of mental health services, i.e., inpatient unit at admission and readmission in the follow-up, were associated with needs at the follow-up in model 1, they were no longer significant in model 2. The needs reported by the Brazilian patients were mainly predicted by socioeconomic indicators observed in the early stages of the disorder (first admission), such as living arrangements, occupational status, and years of education.

Differently from our initial hypothesis, the total number of needs reported by the patients was, in general, lower than those reported in both wealthier countries (14,20,28) and LMICs (23,29,30). A possible explanation for these discrepancies can be related to the cultural background since previous data suggest differences in the report of unmet needs depending on the ethnic heritage (15). Considering the low socioeconomic and education levels of our patients, we cannot rule out that they may be less likely to be aware of their own needs.

Another explanation can be related to the fact that Ribeirão Preto is a city with a public mental health services network with clear catchment areas and established rules and flows for referrals and discharges, regarding both mental and general health services, as well as with regular and periodic meetings of service representatives to discuss cases and policies (25). Accordingly, we found that needs that rely on mental health care (such as psychotic symptoms and psychological distress) and physical health were considered as met by most patients. This explanation may also be reinforced by the rates of adherence to community-based services observed in our sample. Considering the limitations of public policies in the country and the small budget allocated for mental health actions (31), the 58.3% adherence rate found in the present study in at least four years of follow-up can be considered satisfactory, when compared with data from other countries (32,33).

On the other hand, welfare benefits were the most cited unmet need. Accordingly, money was also reported as an unmet need, although less often. This can be related to poor social support from public policies, which was previously observed in other LMICs (30), but not in wealthy countries (14,34), where social security governmental policies are more consolidated. Another explanation for our results can also be due to the patients' and caregivers' lack of knowledge about the Brazilian laws and the eligibility criteria for receiving welfare benefits, as previously reported (27).

Household skills, although one of the most present needs, were rarely unmet. This is probably linked to the predominant family structure in Brazil, where sick people are frequently cared for at home by their relatives (35). On the other hand, needs that relate to a closer relationship with people outside the family (such as daytime activities and company) were more often unmet, showing the importance to effectively implement public policies that address the social integration of people with mental disorders (36), something that has been done in only a partial way in Brazil to date (5,6). Patients living alone had significantly more unmet needs than those living with a partner, which reinforces that family is the main support source to meet needs (35).

Although not statistically significant, we also found a higher rate of unmet needs reported by patients first admitted due to substance use disorders. This result can be related to the higher levels of comorbidities, such as other mental disorders and other medical conditions, of people with substance use problems, and the absence of appropriate and integrated care of all the needs of these patients. However, it is also known that patients with substance use problems have low adherence to treatment and repeated relapses (37). For instance, a cross-sectional survey among homeless adults in Addis Ababa (Ethiopia) found patients with 80-100% of unmet needs across all CAN's domains, the majority of them with substance use-related problems (38).

Although mortality was not within the scope of our study, the high percentage of deaths (8.2%; 44 deaths in 538) in our four-year follow-up period drew our attention, since this rate of mortality is above thirteen times the general Brazilian mortality (https://brasilemsintese.ibge.gov.br/populacao/taxas-brutas-de-mortalidade.html), a finding that goes in the same direction as in other countries (39). Further studies to better assess the rates of mortality in Brazilian psychiatric patients are needed.

Our results should be considered in view of several study limitations. One of them is the number of dropouts. The most common cause of loss was failure to locate addresses, either because they did not exist or because patients no longer lived at the addresses they had reported four years earlier. There were possibly more non-adherent patients in the group that could not be found. It is worth mentioning that, unlike other countries, Brazil has no unified data storage system, making it more difficult to conduct studies of this type. Caution is needed since the high dropout rate can prevent generalizing our findings. Even though a dropout of 42.3% during at least four years may be considered acceptable, the adherence of psychiatric patients to follow-up studies is modest (40). However, the missing patients were younger and mainly with substance use-related problems, which is the group with the most unmet needs observed in our sample. Patients with substance use problems are more prone to being arrested and have a greater risk of death than the general population (37). Still, we cannot rule out that the missing patients had better global functioning than those interviewed. Another limitation is that our database is of patients first admitted a few years ago and comparisons with current patients should be performed cautiously. However, we believe that our results are of current relevance since very few studies on the needs of psychiatric patients have been carried out in Brazil and other LMICs. In addition, we showed an association of sociodemographic and clinical factors with psychiatric patients' needs that can contribute to the definition of current mental health policies. Finally, we must recognize that we considered the main diagnosis registered in the medical records, based on non-structured interviews, and no standard instrument was used for the confirmation of the diagnosis.

On the other hand, we examined all psychiatric diagnoses, not just a specific group, and considered patients whose entrance in the mental health system was due to a severe psychiatric condition. Assessing patients in the early stages of mental disorders allowed the control of possible confounders, such as the effect of lengthy exposition to psychotropic drugs, insufficient adherence to treatment, and appearance of comorbidities, which can also be related to met and unmet needs. We also searched the eligible patients in the community, including patients who were not included in the local mental health network, bringing information about patients who did not seek medical care.

In conclusion, our study demonstrated that socioeconomic indicators, such as living alone or with relatives, low education, and unemployment, were the best predictors of needs reported by Brazilian patients at least four years after their first psychiatric admission, independent of the diagnosis. While needs related to direct support of family and mental health services were considered to be met by most patients, people without such support showed a higher percentage of unmet needs. Moreover, the absence of access to welfare benefits was the most critical unmet need reported. Taken together, these factors point to the great socioeconomic disadvantages of these patients, suggesting a significant impact of the global social context and social security policies in their ability to achieve or restore acceptable levels of social independence and quality of life. Of particular concern was the higher levels of unmet needs among those with the diagnosis of substance use-related problems. Public policies aimed at improving the quality of the care and the effectiveness of the treatment of such serious health problems are urgently needed.

## Supplementary Material

Click here to view [pdf].
